# Supplementary material for: Association between oral microbiome and seven types of cancers in East Asian population: a two-sample Mendelian randomization analysis
Source: Front Mol Biosci. 2023 Nov 21;10:1327893. doi: 10.3389/fmolb.2023.1327893 (PMC10702768; doi:10.3389/fmolb.2023.1327893)
Supplement: Supplementary file 1 [file DataSheet1.ZIP › Supplementary Materials/MR plots of saliva/saliva═╝/colorectal cancer/pheno.3170_to_colorectal cancer_forest.pdf]

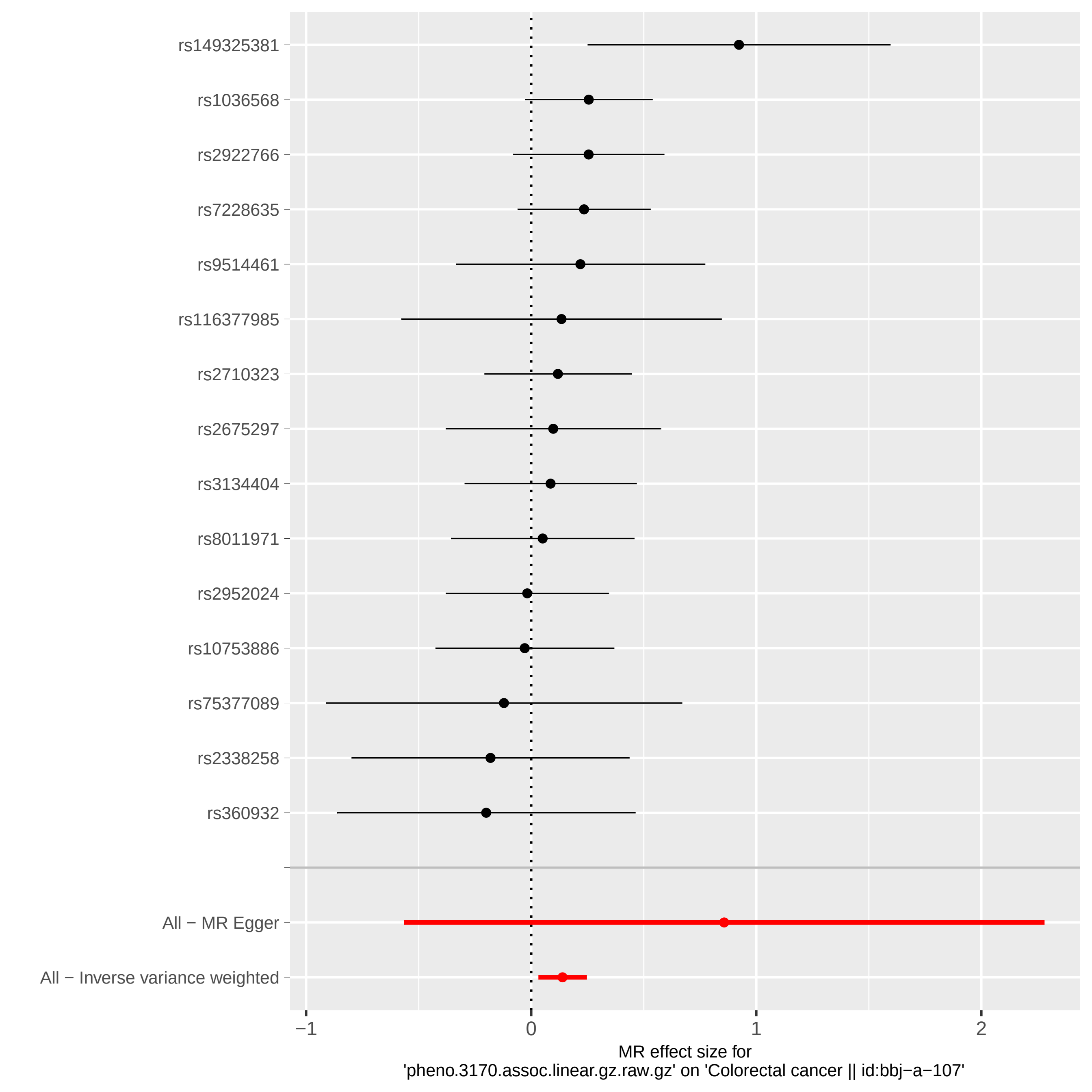
rs149325381
rs1036568
rs2922766
rs7228635
rs9514461
rs116377985
rs2710323
rs2675297
rs3134404
rs8011971
rs2952024
rs10753886
rs75377089
rs2338258
rs360932
All − MR Egger
All − Inverse variance weighted
−1
0
1
2
MR effect size for
'pheno.3170.assoc.linear.gz.raw.gz' on 'Colorectal cancer || id:bbj−a−107'
